# Combinatory Multifactor Treatment Effects on Primary Nanofiber Oligodendrocyte Cultures

**DOI:** 10.3390/cells8111422

**Published:** 2019-11-12

**Authors:** Lukas S. Enz, Thomas Zeis, Annalisa Hauck, Christopher Linington, Nicole Schaeren-Wiemers

**Affiliations:** 1 Neurobiology, Department of Biomedicine, University Hospital Basel, University Basel, Zentrum für Lehre und Forschung, 4031 Basel, Switzerland; lukas.enz@unibas.ch (L.S.E.); thomas.zeis@unibas.ch (T.Z.); annalisa.hauck@unibas.ch (A.H.); 2 Institute of Infection, Immunity and Inflammation, College of Medical Veterinary and Life Sciences, University of Glasgow, Glasgow G12 8TA, UK; christopher.linington@glasgow.ac.uk

**Keywords:** oligodendrocyte, myelin, remyelination, screening, nanofibers, multiple sclerosis

## Abstract

Multiple sclerosis (MS) is a chronic inflammatory demyelinating and neurodegenerative disease of the central nervous system. Neurological deficits are attributed to inflammatory demyelination, which compromises axonal function and survival. These are mitigated in experimental models by rapid and often complete remyelination of affected axons, but in MS this endogenous repair mechanism frequently fails, leaving axons increasingly vulnerable to the detrimental effects of inflammatory and metabolic stress. Understanding the molecular basis of remyelination and remyelination failure is essential to develop improved therapies for this devastating disease. However, recent studies suggest that this is not due to a single dominant mechanism, but rather represents the biological outcome of multiple changes in the lesion microenvironment that combine to disrupt oligodendrocyte differentiation. This identifies a pressing need to develop technical platforms to investigate combinatory and/or synergistic effects of factors differentially expressed in MS lesions on oligodendrocyte proliferation and differentiation. Here we describe protocols using primary oligodendrocyte cultures from Bl6 mice on 384-well nanofiber plates to model changes affecting oligodendrogenesis and differentiation in the complex signaling environment associated with multiple sclerosis lesions. Using platelet-derived growth factor (PDGF–AA), fibroblast growth factor 2 (FGF2), bone morphogenetic protein 2 (BMP2) and bone morphogenetic protein 4 (BMP4) as representative targets, we demonstrate that we can assess their combinatory effects across a wide range of concentrations in a single experiment. This in vitro model is ideal for assessing the combinatory effects of changes in availability of multiple factors, thus more closely modelling the situation in vivo and furthering high-throughput screening possibilities.

## 1. Introduction

Multiple Sclerosis (MS) is a chronic inflammatory, demyelinating disease of the central nervous system (CNS) with diverse clinical presentations and a heterogeneous histopathology. It is suggested that demyelination and failure of remyelination lead to axonal degeneration and functional impairment. This axonal pathology is the underlying cause of disability in patients with MS and is attributed to the detrimental effects of inflammatory demyelination on the functional and structural integrity of affected axons [1,2]. In this context, demyelination not only disrupts axonal conduction per se but also disrupts metabolic support provided via the myelin sheath whilst simultaneously enhancing axonal susceptibility to damage by inflammatory mediators, a combination of effects predicted to exacerbate axonal loss and result in irreversible neurological deficits. These effects of demyelination on axonal health are mitigated in animal models by rapid and often complete remyelination by oligodendrocytes derived from an endogenous pool of oligodendrocyte progenitor cells (OPCs). However, in MS, this repair mechanism frequently fails, leaving affected axons increasingly vulnerable to inflammatory and metabolic stress [3].

Why remyelination fails in MS remains poorly understood, but it is attributed to changes in the lesion microenvironment that compromise OPC recruitment, survival and/or differentiation, and axon function [4,5,6,7,8,9], which are compounded by factors including disease chronicity [10] and factors not related to the disease, such as gender and age [11].

Developmental myelination is under tight spatial and temporal control and is coordinated by factors including neuronal activity, changes affecting growth factor availability, and stage specific expression of their receptors within the oligodendrocyte lineage. It is assumed that remyelination recapitulates many features of this complex developmental program [12]. It is therefore not surprising that remyelination can be disrupted experimentally by many different mechanisms, including inappropriate re-expression of developmental cues and continuing presence of myelin debris as well as changes affecting growth factor availability, progenitor cell migration, and composition of the extracellular matrix [13]. Strong circumstantial arguments can be made for each of these mechanisms contributing to remyelination failure in MS, but their relative importance is unclear. To resolve this question, several groups performed comparative transcriptomic studies on normal-appearing white matter, active inflammatory demyelinating lesions, remyelinating lesions, and inactive chronically demyelinated lesions with the aim of identifying specific molecules or signaling pathways that may provide targets for treatment strategies designed to enhance remyelination [14,15,16,17]. However, these studies have failed to identify any single factor or pathway consistently associated with remyelination failure. Instead they demonstrate there is significant intra-study and -lesion heterogeneity with regard to the expression of genes predicted to influence oligodendrogenesis and myelination between different lesion types. An observation that has led us to speculate failure of remyelination is the net result of dysregulation of multiple pathways.

To understand the consequences of this heterogeneity, an experimental platform is required in which we can rapidly assess concentration dependent effects of multiple factors on OPC proliferation, differentiation, and myelination. Primary co-culture systems are suitable to investigate specific questions on oligodendrocyte development and myelination, but due to the complexity of these systems, direct effects on proliferation, differentiation, and myelination can hardly be discerned from indirect effects. Quantitative approaches are often handicapped due to the cell heterogeneity in these co-culture systems. In 2012, the research group of Jonah Chan demonstrated oligodendrocytes are capable of myelinating engineered nanofibers [18]. This technique has enormous potential to evaluate the effect of single molecules on oligodendrocyte myelination, and to identify potential therapeutic targets [19].

To demonstrate the effectiveness of this platform we focused on four factors differentially regulated in MS lesions that influence OPC proliferation, differentiation, and myelination: platelet-derived growth factor subunit A dimer (PDGF–AA [20]), fibroblast growth factor 2 (FGF2 [15,21]), bone morphogenetic protein 2 (BMP2 [22]), and bone morphogenetic protein 4 (BMP4 [22,23]). We were able to show that our model system is an efficient way of screening effects in a multifactor treatment paradigm. A major application of this model system may be the modeling of the complex environment in a demyelinated lesion.

## 2. Materials and Methods

### 2.1. Oligodendrocyte Cell Culture

Primary oligodendrocyte cultures were prepared from post-natal day 0–2 C57Bl/6 mice, as described before [24]. Briefly, forebrains were collected aseptically and after removal of the meninges, the cortices were triturated 35 times through a 1 mL pipet in “DBGFP”medium (Table 1) and filtered through a 70 μm cell strainer. Cells were grown in suspension culture in DBGFP at 37 °C, 5% CO2, and 95% air in DBGFP to generate oligospheres. After two weeks, cultures from multiple donors were pooled, centrifuged at 300× *g* for five minutes, and resuspended in plating medium (Table 1). After gentle trituration through a G27 needle, cells were plated at a density of 10,000 oligodendrocytes/well in 384-well nanofiber plates (Z694568–1EA, Sigma–Aldrich Chemie GmbH, Buchs, Switzerland) coated with poly–l–lysine (P4707–50ML, Sigma–Aldrich Chemie GmbH, Buchs, Switzerland).

### 2.2. Immune Fluorescence Stainings

Immunofluorescent stainings were either performed with 4′,6-diamidin-2-phenylindol (DAPI) and against galactosylceramide (O1), platelet-derived growth factor alpha (PDGFRa) and myelin basic protein (MBP) or with DAPI and against myelin oligodendrocyte glycoprotein (MOG), oligodendrocyte lineage factor 2 (OLIG2), and glial fibrillary acidic protein (GFAP) (Table 2).

First, for staining of living cells (O1 staining), the cultured cells were blocked in 10% fetal bovine serum in DMEM/F12 medium at 37 °C for 30 min, and thereafter the cells were incubated for 30 min with O1 supernatant at room temperature. All wells were then washed twice with phosphate buffered saline (PBS), and fixed with 4% paraformaldehyde for 15 min at room temperature. Thereafter, the cells were washed three times with PBS for five minutes and incubated for 1 h with blocking solution (5% bovine serum albumin, 1% normal donkey serum, 0.2% Triton-X100 in PBS). Primary antibodies were diluted in blocking solution and applied overnight at 4 °C (Table 2). The cells were washed three times with PBS (15 min each at room temperature). The secondary antibody (Table 2) was then applied for one hour in PBS with DAPI at room temperature. The cells were washed three times with PBS again and then rinsed twice with tap water and covered with ibidi mounting medium (ibidi GmbH, Gräfelfing, Germany, Cat. No. 50001).

### 2.3. Image Analysis

**Figure 1:**
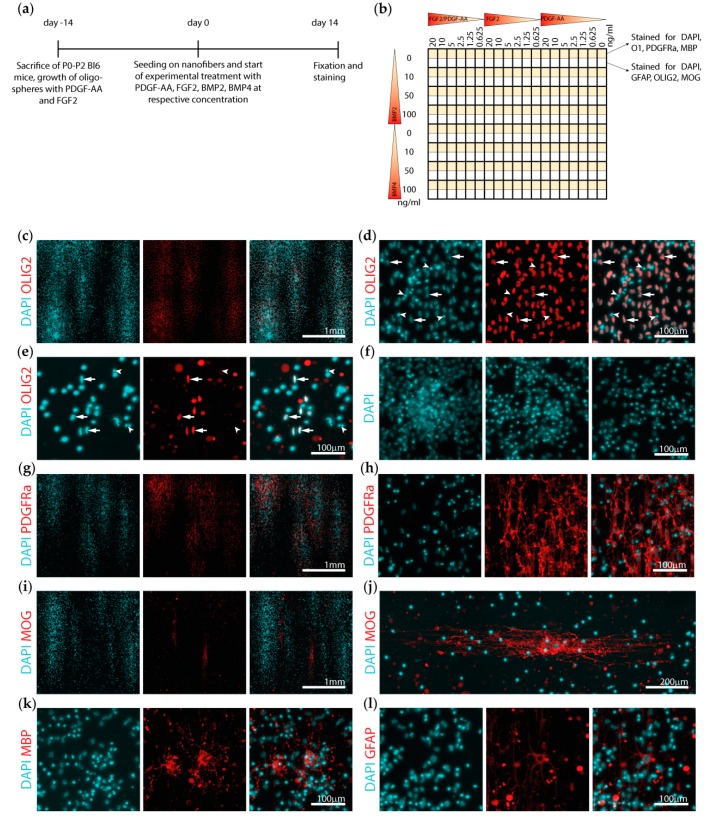
Experimental set up and cell culture characterization. (**a**) Illustration of the experimental timeline showing the sacrifice of the mice, two-week growth of the oligospheres, and plating on the nanofibers. (**b**) Schematic drawing of the experimental set-up of the 384-nanofiber plates. (**c**–**l**) Representative images of the different stainings. (**c**) The cells proliferated in clusters growing along the nanofibers. (**d**) Most of the nuclei (stained with DAPI) colocalized with the oligodendrocyte lineage marker OLIG2 (arrows, well treated with 20 ng/mL platelet-derived growth factor subunit A dimer (PDGF–AA) and fibroblast growth factor 2 (FGF2). (**e**) Note, some nuclei showed a dense and sometimes fragmented morphology and were not positive for oligodendrocyte lineage factor 2 (OLIG2) (arrowheads, (**d** and **e**)). This was more pronounced in the wells with less PDGF–AA and FGF2 and more bone morphogenetic protein 2 (BMP2) (well treated with 0.625 ng/mL PDGF–AA and FGF2). (**f**) Dense cell clusters were not eligible for accurate counting of the nuclei. (**g**, **h**, and **k**) PDGFRa and MBP were mainly expressed within the clusters, while (**i** and **j**) the larger MOG positive cells were usually situated in proximity but not within the clusters. (**l**) Possible contamination with glial fibrillary acidic protein (GFAP) positive astrocytes was very rare, with most wells showing no staining for GFAP.

384-well plates were imaged using a Nikon Ti2 microscope (Nikon, Tokyo, Japan) fitted with a Prime 95B camera (Teledyne Photometrics, Tucson, AZ, USA). The center of each well was scanned (6 × 6 fields of view) with z-stacks over 27.2 µm with 1.7 µm per slice and a resolution of 0.28 micrometer per pixel. This resulted in images of 2.37 × 2.37 mm or about 51% of the total well area. For the illustrative images of Figure 1, an Olympus IX83 microscope was used. All image analysis was then performed with ImageJ (Version 1.51s, FIJI distribution, NIH, Bethesda, MD, USA). Stacks were first compressed to single images with Z-Project by maximum intensity. For quality control, every DAPI image was reviewed for proper focusing and wells were rescanned if more than 5% of the image was out of focus. All images were then converted to 8-bit images and thresholded at values of 125/255 (DAPI), 160/255 (PDGFRa) or 75/255 (MBP). The percentage of pixels above the threshold was considered as the percent of area positive for the respective marker and used for further analysis.

### 2.4. Statistical Analysis

All statistical analysis was performed using R (R Development Core Team, Vienna, Austria, 2010). The experiments were performed on three 384-well plates, leading to a total of six wells per treatment condition for DAPI and three wells per treatment condition for all other stainings (Figure 1B). Each plate was normalized to the average measurement per plate, the measurements and treatments were log-transformed, and a pseudo count was added to the treatment values to avoid log of zero. Obvious outliers were removed from the analysis and data from all three plates was pooled for the analysis. Two different linear models were fitted: one with PDGF–AA, FGF2, and BMP2 and all possible interaction terms as independent variables and the other with BMP4 instead of BMP2. *p*-values smaller than 0.01 were considered to be statistically significant.

### 2.5. Data Availability

All original greyscale microscopy images used for image analysis are deposited on the BioStudies database of the European Bioinformatics Institute (EMBL-EBI, Hinxton, Cambridge, UK, accession number S-BIAD10, link: https://www.ebi.ac.uk/biostudies/BioImages/studies/S-BIAD10). All original image analysis data may be found in the supplements (Supplementary Data 1).

## 3. Results

### 3.1. The Nanofiber Cell Cultures Give Rise to Highly Pure Proliferating and Differentiating Oligodendrocytes

Primary mouse oligodendrocytes were cultivated as spheres and plated on three 384-well nanofiber plates for 14 days with PDGF–AA and FGF2 in combination or independently concentrated at 0, 0.625, 1.25, 2.5, 5, 10, or 20 ng/mL and BMP2 or BMP4 concentrated at 0, 10, 50, or 100 ng/mL (Figure 1a,b). The cells were then stained for PDGFRa, O1, MBP, OLIG2, MOG, or GFAP and scanned with a microscope. A total of 911 of 912 wells were correctly focused and could be used for further analysis.

Image inspection revealed clusters of cells oriented along the nanofibers expressing the oligodendrocyte lineage marker OLIG2 (Figure 1c, arrows). Some nuclei did not show colocalization with OLIG2, these nuclei were however most often dense or fragmented, suggestive of cell death (Figure 1d, arrowheads). This phenomenon was more pronounced in the wells treated without or with only relatively low concentrations of PDGF–AA and FGF2 (Figure 1e). Initial efforts to quantify the number of cells by counting the DAPI nuclei demonstrated cell numbers between 3000 (e.g., upon 100 ng/mL BMP2 treatment) and 12,000 cells (e.g., upon 20 ng/mL PDGF-AA and FGF2 treatment) per imaged area to be present, clearly indicating that proliferation and cell death occurred after plating of the initial 10,000 cells per well. However, in particular, the dense cell clusters were not eligible for automated cell counting, and thus a more simplified approach of evaluating the area stained for DAPI was chosen as a surrogate marker for the number of cells present (Figure 1f).

PDGFRa was mostly expressed in the densely populated areas, with only little expression between the clusters (Figure 1c). Expression of the myelin protein MOG was most often detected at the border of the dense cell clusters (Figure 1c), whereas MBP signal was both detected in smaller cells within the clusters and in larger cells between the clusters (Figure 1c). Staining for astrocytes with GFAP revealed only very few astrocytes being present, while most wells showed no GFAP-positive cell (Figure 1c).

### 3.2. Cell Density Is Strongly Increased by Platelet-Derived Growth Factor Subunit A Dimer (PDGF–AA), While Fibroblast Growth Factor 2 (FGF2) Potentiated the Effect of PDGF–AA and Bone Morphogenetic Protein 2 (BMP2) Overrides the Effects of PDGF–AA

**Figure 2:**
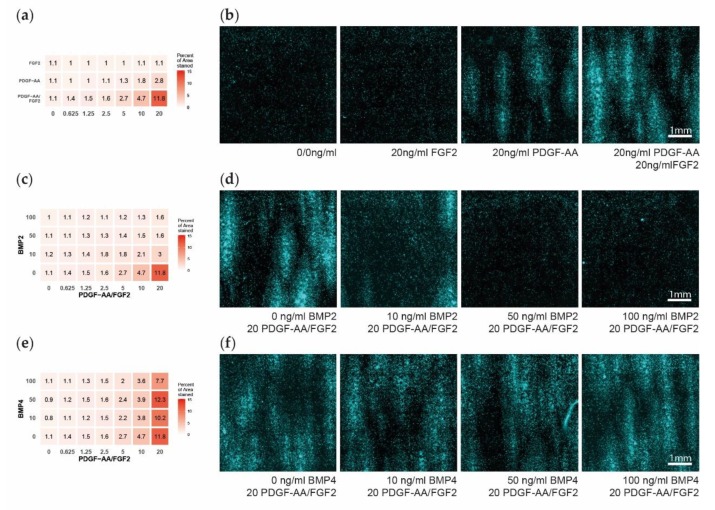
Oligodendrocyte progenitor cells (OPC) cell density after 14 days of multifactor treatment. (**a**) Heatmap showing the percent of area positive for DAPI, as a measure for OPC cell density, dependent on the platelet-derived growth factor subunit A dimer (PDGF–AA) and fibroblast growth factor 2 (FGF2) treatment. (**b**) Representative microscopy images showing the DAPI signal upon different PDGF–AA and FGF2 treatments. (**c**) Heatmap showing the percent of area positive for DAPI dependent on the PDGF–AA/FGF2 and bone morphogenetic protein 2 (BMP2) treatment. (**d**) Representative microscopy images of PDGF–AA and FGF2 concentrated at 20 ng/mL and with BMP2 levels of 0–100 ng/mL. (**e**) Heatmap showing the percent of area positive for DAPI dependent on the PDGF–AA/FGF2 and BMP4 treatment. (**f**) Representative microscopy images of PDGF–AA and FGF2 concentrated at 20 ng/mL and with BMP4 levels of 0–100 ng/mL.

After 14 days in culture, the OPCs have proliferated to cover on average 1.7% of the area with DAPI (range: 0.5–19.3%). We detected a gradient of increased cell density with rising levels of PDGF–AA as detected by the area stained positive for DAPI, independent of the FGF2 concentration (Figure 2a,b). FGF2 in absence of PDGF–AA did not show an effect on cell density, but enhanced cell density with increasing concentrations if a certain amount of PDGF–AA was simultaneously present (Figure 2a,b). The effect of PDGF–AA on cell density and the interaction with FGF2 was statistically significant (Table 3).

Already, relatively low concentrations of 10 ng/mL of BMP2 over 14 days were able to nullify the cell density effect of PDGF–AA and FGF2, with higher concentrations of BMP2 showing no additional effect. BMP2 showed no effect if PDGF–AA was not present, and the calculated positive main effect of BMP2 from the linear model should not be interpreted due to the detected interactions with PDGF–AA and FGF2 (Figure 2c,d, Table 3). BMP4 showed no effect on cell density for all concentrations tested (Figure 2e,f, Table 3).

### 3.3. Early Differentiation Is Promoted by Platelet-Derived Growth Factor Subunit A Dimer (PDGF–AA), Enhanced by Fibroblast Growth Factor 2 (FGF2) and Inhibited by Bone Morphogenetic Protein 2 (BMP2)

**Figure 3:**
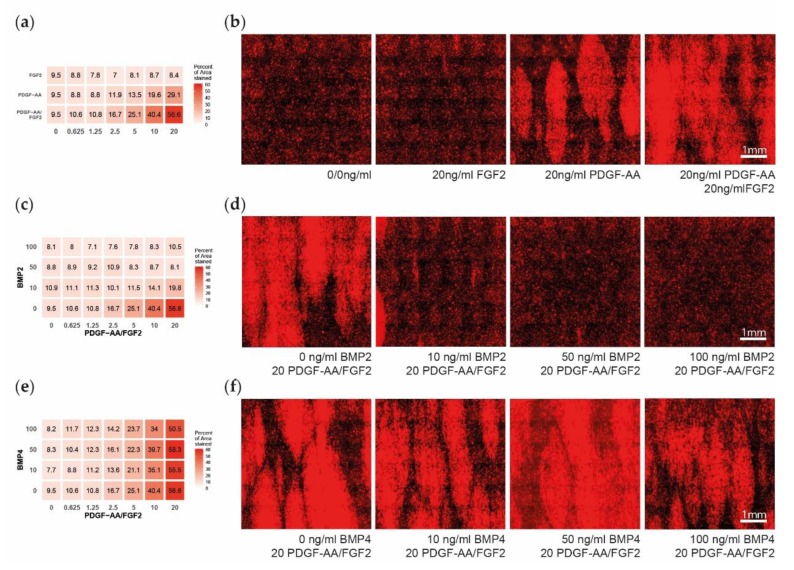
Early oligodendrocyte progenitor cells (OPC) differentiation after 14 days of multifactor treatment. (**a**) Heatmap showing the percent of area positive for platelet-derived growth factor alpha (PDGFRa), as a measure for early OPC differentiation, dependent on the platelet-derived growth factor subunit A dimer (PDGF–AA) and fibroblast growth factor 2 (FGF2) treatment. (**b**) Representative microscopy images showing the PDGFRa expression upon different PDGF–AA and FGF2 treatments. (**c**) Heatmap showing the PDGFRa expression in dependency of the PDGF–AA/FGF2 and bone morphogenetic protein 2 (BMP2) treatment. (**d**) Representative microscopy images of PDGF–AA and FGF2 concentrated at 20 ng/mL and with BMP2 levels of 0–100 ng/mL. (**e**) Heatmap showing the PDGFRa expression in dependency of the PDGF–AA/FGF2 and BMP4 treatment. (**f**) Representative microscopy images of PDGF and FGF2 concentrated at 20 ng/mL and with BMP4 levels of 0–100 ng/mL.

14 days after seeding the cells have differentiated to cover an average of about 43.3% with PDGFRa signal (range: 28.4–77.8%). A gradient of PDGFRa expression is detected with increasing levels of PDGF–AA independent of the FGF2 concentration (Figure 3a,b). With increasing levels of FGF2, a gradient of PDGFRa signal is only detected if minimal concentrations of PDGF–AA are present. Both the effect of PDGF–AA and the interaction of PDGF–AA and FGF2 reached statistical significance (Table 3).

This gradient disappears with increasing levels of BMP2 dose dependent between 0–50 ng/mL with only minimal additional effect of 100 ng/mL BMP2 (Figure 3c,d). While the main effect of BMP2 did not reach significance, the interaction of BMP2 with PDGF–AA and with PDGF–AA and FGF2 reached statistical significance (Table 3). BMP4 did not show any effect on PDGFRa expression, independent of its concentration (Figure 3e,f, Table 3).

### 3.4. Late Differentiation is Unaffected by Platelet-Derived Growth Factor Subunit A Dimer (PDGF–AA), Fibroblast Growth Factor 2 (FGF2), Bone Morphogenetic Protein 2 (BMP2) and BMP4

**Figure 4:**
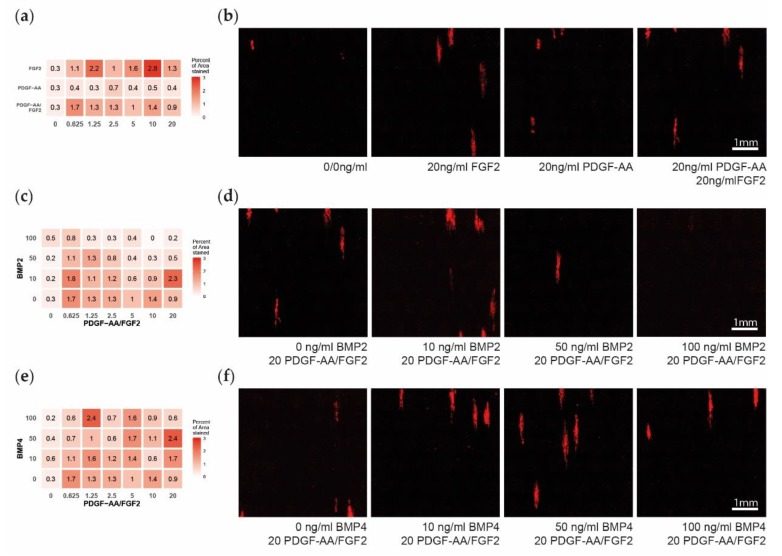
Late oligodendrocyte progenitor cells (OPC) differentiation after 14 days of multifactor treatment. (**a**) Heatmap showing the influence of platelet-derived growth factor subunit A dimer (PDGF–AA) and fibroblast growth factor 2 (FGF2) on late differentiation measured as percent of area positive for myelin basic protein (MBP). (**b**). Representative microscopy images showing the MBP expression. (**c**) Heatmap showing the influence of bone morphogenetic protein 2 (BMP2) on MBP expression. (**d**) Representative microscopy images of PDGF–AA and FGF2 concentrated at 20 ng/mL and with BMP2 levels of 0–100 ng/mL. (**e**) Heatmap showing the influence of BMP4 on MBP expression. (**f**) Representative microscopy images of PDGF–AA and FGF2 concentrated at 20 ng/mL and with BMP4 levels of 0–100 ng/mL.

The treatments with PDGF–AA, FGF2, BMP2, and BMP4 did not demonstrate statistically significant effects on MBP expression (Figure 4a–e, Table 3). Though BMP2 seemed to inhibit late differentiation with high concentrations, this effect was not linear and did not reach statistical significance (Figure 4c,d). Upon visual investigation, we observed no apparent change in the length, processes, or area of the MBP expressing cells.

## 4. Discussion

Recently, high-throughput screening studies have identified several compounds that promote remyelination [25,26,27,28], including clemastine, which is currently being investigated in a phase II clinical trial in optic neuritis (Clinical trials identifier NCT02521311). However, these screens focused on screening individual factors under culture conditions that do not replicate the complex pathologic environment of an MS lesion. To address this question, we elected to build on existing technologies to develop a platform in which we could rapidly screen for multiparametric effects that mimic the lesion microenvironment and its effects on cells of the oligodendrocyte lineage.

We successfully established a cell culture system in which highly purified oligodendrocytes myelinate nanofibers in 384-well nanofiber plates to reliably and efficiently quantify the effects of multi-factor treatments on oligodendrocyte development and myelination. We implemented a standardized operating procedure for cell culturing, imaging, and image analysis that optimizes reproducibility and mapped combinatory, concentration-dependent effects of PDGF–AA, FGF2, BMP2 and BMP4 on oligodendrocytes and myelination.

PDGF–AA is known to be essential for oligodendrocyte generation and survival [29] and differentiation [30]. We detected a positive effect of PDGF–AA on the number of cells as reported before [31,32,33]. We also detected a positive effect of PDGF–AA on early OPC differentiation into PDGFRa positive cells. This simultaneous positive effect on differentiation is controversial, however, as a factor promoting proliferation effectively keeps a cell in the cell-cycle, while promoting differentiation requires the cell to exit the cell-cycle. Cell culture experiments have demonstrated that proliferation may cause mechanical restraint, which then induces OPC differentiation indirectly [34]. We interpret the expression of PDGFRa mainly within the more densely populated clusters in this manner. In our cell culture system, FGF2 showed no effect on cell density and early OPC differentiation in absence of PDGF–AA. However, FGF2 showed an increasing effect on both cell density and differentiation with rising levels of PDGF–AA being present, thus enhancing the effect of PDGF–AA. As suggested by our own observations, FGF2 has been shown to not be essential for OPC development, in contrast to PDGF–AA [30]. FGF2 is further known to stimulate OPC proliferation and migration and to increase oligodendrocyte process elongation [35,36], but also to have a negative effect on differentiation [30] and myelination [37], by keeping the OPC in the cell-cycle.

BMPs were reported to inhibit OPC proliferation, oligodendrocyte maturation, and myelin protein expression and to push OPCs into the astrocyte lineage, if thyroid hormone is present as well [38,39,40,41]. In line with these previous data, our results demonstrate a strong reduction of OPC cell density and differentiation caused by BMP2. We further demonstrated that BMP2 effectively overwrites the effect of PDGF–AA and FGF2 already at low concentrations, irrespective of the concentrations of PDGF–AA and FGF2. As only very few GFAP expressing cells developed within our culture systems, we did not expect and did not detect an increase in astrocytes when adding BMP2. In our study, BMP4 had no effect on cell density and differentiation. This may be explained by the fact that BMP4 only acts in a certain time frame of OPC development, mainly during the GalC positive phase. In our culture system, this phase may have already been passed during the two weeks the cells were kept as oligospheres prior to seeding on the nanofibers.

As our system was intended to model the signaling environment of a demyelinated lesion by expressing factors known to be expressed there, we did not expect to detect many cells showing late differentiation. Accordingly, we detected few MBP positive cells, statistically irrespective of the treatment. This may be suggestive of a fate determination prior to treatment beginning, possibly already within the brain or during the oligosphere stage. These cells differentiated remarkably despite high BMP2 levels, which otherwise efficiently inhibit both proliferation and early differentiation. Our treatments however are not optimized for assessing late differentiation and myelination, as this would require for example a withdrawal of PDGF–AA and FGF2, which keep the OPCs within proliferation.

Even though the dysregulation of the factors tested here has been demonstrated on RNA and protein level in MS lesions, there is currently no data available concerning the local concentrations of these factors. While the concentrations of PDGF–AA and FGF2 have been measured in the cerebrospinal fluid [42,43,44], BMP levels have so far only been determined within blood serum samples of MS patients [45]. However, this data cannot easily be extrapolated to the local situation within lesions, where concentrations could potentially reach much higher levels.

We may conclude that our cell culture system is suitable for studying more complex effects of multifactor treatments on oligodendrocytes. This approach enables to better model the signaling environment of MS lesions, where the decision is made, whether lesions are chronically demyelinated or remyelination and thus functional recovery may occur. To this end, any improved mapping and quantification of the factors present in vivo in MS lesions may improve the in vitro model system. This model may then serve as an alternative platform to screen novel therapeutic approaches in the ongoing research on remyelination therapy.

## Figures and Tables

**Table 1:** Composition of used cell culture media.

	Product	Product Number	Company	Dilution/Concentration
**DBGFP:**	Dulbecco’s Modified Eagle’s Medium/Nutrient Mixture F-12 Ham with (4-(2-hydroxyethyl)-1-piperazineethanesulfonic acid) (HEPES)	31330095	Gibco/ThermoFisher Scientific, USA	-
	B–27 Supplement (50×)	17504001	Gibco/ThermoFisher Scientific, USA	1:50
	l–Glutamine (200 mM)	25030024	ThermoFisher Scientific, USA	1:100
	Fibroblast growth factor 2 (FGF2)	100–18B	PeproTech EC, Ltd., UK	20 ng/mL
	Platelet-derived growth factor (PDGF)	100–13A	PeproTech EC, Ltd., UK	20 ng/mL
	Antibiotic–Antimycotic	15240062	Gibco/ThermoFisher Scientific, USA	1:100
**Plating:**	DBGFP medium without PDGF–AA and FGF2.			
**Treatment:**	DBGFP medium without PDGF–AA and FGF2 and one/two/three of the following:			
	PDGF–AA	100–13A	PeproTech EC, Ltd., UK	0–20 ng/mL
	FGF2	100–18B	PeproTech EC, Ltd., UK	0–20 ng/mL
	Bone morphogenetic protein 2 (BMP2)	120–02	PeproTech EC, Ltd., UK	0–100 ng/mL
	BMP4	315–27	PeproTech EC, Ltd., UK	0–100 ng/mL

**Table 2:** Dyes and antibodies used for the stainings.

Dye/Antibody	Type/Species	Company	Cat. Nr.	Dilution
4′,6-Diamidin-2-phenylindol (DAPI)	-	Sigma Aldrich		1:15,000
O1	Monoclonal/Mouse	Kindly provided by Prof. M. Schwab, Zürich, CH	D9542-10 mg	1:250
Platelet derived growth factor alpha (PDGFRa)	Polyclonal/Rabbit	Kindly provided by Prof. William B. Stallcup, La Jolla, CA, US		1:4000
Myelin basic protein (MBP)	Monoclonal/Rat	Merck Millipore		1:250
Myelin oligodendrocyte glycoprotein (MOG)	Monoclonal/Mouse	Kindly provided by Prof. R. Reynolds, London, UK	MAB386	1:250
Oligodendrocyte linear factor 2 (OLIG2)	Polyclonal/Rabbit	Merck Millipore	Clone Z12	1:2000
Glial fibrillary acidic protein (GFAP)	Polyclonal/Chicken	Aves Labs	AB9610	1:2000
dk–a–m–488	Monoclonal/Donkey	Jackson ImmunoResearch	AB_2313547	1:700
dk–a–rb–594	Monoclonal/Donkey	Jackson ImmunoResearch		1:700
dk–a–rt–647	Monoclonal/Donkey	Jackson ImmunoResearch	715-545-140	1:700
dk–a–ck–647	Monoclonal/Donkey	Jackson ImmunoResearch	711-585-152	1:700
			712-605-150	
			703-605-155	

**Table 3:** Significant estimates and *p*-values derived from the linear model.

	Factor	DAPI	*p*-Value	Platelet-Derived Growth Factor Alpha (PDGFRa)	*p*-Value
BMP2 Models	platelet-derived growth factor subunit A dimer (PDGF–AA)	0.26	<10^−8^	0.49	<10^−13^
	Fibroblast growth factor 2 (FGF2)		ns		ns
	PDGF–AA:FGF2	0.19	<10^−15^	0.11	<10^−5^
	Bone morphogenetic protein 2 (BMP2)	0.11	<10^−5^		ns
	PDGF–AA:BMP2	−0.05	<10^−7^	−0.08	<10^−6^
	FGF2:BMP2		ns		ns
	PDGF–AA:FGF2:BMP2	−0.02	<10^−8^	−0.02	<10^−3^
BMP4 Models	PDGF–AA	0.21	<10^−5^	0.41	<10^−8^
	FGF2		ns		ns
	PDGF–AA:FGF2	0.18	<10^−15^	0.11	<10^−5^
	BMP4		ns		ns
	PDGF–AA:BMP4		ns		ns
	FGF2:BMP4		ns		ns
	PDGF–AA:FGF2:BMP4		ns		ns

The colon “:” depicts interaction terms between the factors, ns: not significant.

## Supplementary Materials

The following are available online at https://www.mdpi.com/2073-4409/8/11/1422/s1, Data S1: Image analysis data.
